# Drug Solubility Enhancement through the Preparation of Multicomponent Organic Materials: Eutectics of Lovastatin with Carboxylic Acids

**DOI:** 10.3390/pharmaceutics11030112

**Published:** 2019-03-09

**Authors:** Andrea Mariela Araya-Sibaja, José Roberto Vega-Baudrit, Teodolito Guillén-Girón, Mirtha Navarro-Hoyos, Silvia Lucia Cuffini

**Affiliations:** 1 Laboratorio Nacional de Nanotecnología LANOTEC-CeNAT-CONARE, Pavas 1174-1200, San José, Costa Rica; andrea.araya@gmail.com (A.M.A.-S.); jvegab@gmail.com (J.R.V.-B.); 2 Centro de Investigación y Extensión en Materiales, Escuela de Ciencia e Ingeniería de los Materiales, Tecnológico de Costa Rica, Cartago 159-7050, Costa Rica; tguillen@itcr.ac.cr; 3 Escuela de Química, Universidad de Costa Rica, San Pedro de Montes de Oca, San José 2060, Costa Rica; mnavarro@codeti.org; 4 Laboratorio de Investigación y Tecnología de Polímeros POLIUNA, Escuela de Química, Universidad Nacional de Costa Rica, Heredia 86-3000, Costa Rica; 5 Instituto de Ciência e Técnica, Universidade Federal de São Paulo, São José dos Campos, CEP: 12.223-201 São Paulo, Brazil

**Keywords:** lovastatin, eutectic mixtures, carboxylic acids, multicomponent organic materials, GRAS substances

## Abstract

Lovastatin (LOV) is a drug used to treat hypercholesterolemia. Recent studies have identified its antioxidant effects and potential use in the treatment of some types of cancer. However, the low bioavailability related to its poor water solubility limits its use in solid oral dosage forms. Therefore, to improve the solubility of LOV three eutectic systems of LOV with the carboxylic acids benzoic (BEN), salicylic (SAL) and cinnamic (CIN) were obtained. Both binary phase and Tammann diagrams were constructed using differential scanning calorimetry (DSC) data of mixtures prepared from 0.1 to 1.0 molar ratios. Binary mixtures and eutectics were prepared by liquid-assisted grinding. The eutectics were further characterized by DSC and powder X-ray diffraction (PXRD), Fourier-transform infrared spectroscopy (FT-IR) and scanning electron microscopy (SEM). The LOV-BEN, LOV-SAL and LOV-CIN system formed a eutectic at an LOV mole fraction of 0.19, 0.60 and 0.14, respectively. The systems exhibited improvements in LOV solubility, becoming more soluble by five-fold in the LOV-SAL system and approximately four-fold in the other two systems. Considering that the solubility enhancements and the carboxylic acids used are generally recognized as safe by the U.S. Food and Drug Administration (FDA), the LOV eutectic systems are promising materials to be used in a solubility enhancement strategy for pharmaceutical product formulation.

## 1. Introduction

Eutectic mixtures are multicomponent organic materials formed by two or more crystalline solids that are immiscible in the solid state and miscible in the liquid state [1,2]. Their formation occurs via noncovalent forces [3,4], mainly hydrogen bonding, ionic and van der Waals forces, and aromatic interactions [4]. In general, the melting temperature of the eutectic is lower than the melting temperatures of their parent components [5]. These systems can be considered as intimately physically blended, with high thermodynamic functions, such as free energy, enthalpy and entropy [6], which provide both solubility and dissolution enhancements [7,8]. Furthermore, highly water-soluble carriers and excipients can favorably impact the wettability of the drug, conferring bioavailability improvements [8,9]. Finally, the increased surface area of the components also contributes in dissolution rate increments similar to what occurs with amorphous solids and solid dispersions [6,7,9]. 

Although organic eutectics possess differences in critical properties related to product performance, like solubility, stability, and bioavailability, they have been relatively unexplored compared to salts and cocrystals [10,11]. In the past few years, the interest in eutectics has increased considerably for pharmaceutical applications, because the use of eutectics in pharmaceutical formulations is quite advantageous compared to other strategies for the following reasons: (1) eutectics are inexpensive, easier to produce and scale up [9,12]; (2) they are not considered new chemical entities [9] or a new crystal form [13] (e.g., cocrystal) and consequently do not require clinical trials; and (3) in eutectic systems, the drug and coformer exist as crystalline compounds, which are highly stable compared to amorphous materials [7,9,14].

Lovastatin (LOV) is widely used to control hypercholesterolemia and is a first-line treatment of coronary artery disease and atherosclerosis, which acts by inhibiting hydroxymethyl glutaryl coenzyme A (HMG-CoA) reductase [15]. Inhibitors of HMG-CoA have been shown to reduce oxidative stress [16], which is related to the mechanisms of initiation and development of several diseases [17], including cardiovascular, neurological, endocrine and gastric disorders, and carcinogenesis and tumor progression [17,18]. In particular, it has been determined that LOV has antioxidant properties and shows an effect on oxidative stress [19]. Recent studies have shown that LOV can induce tumor cell apoptosis [20], inhibit the mevalonate pathway in breast cancer [21], is considered as a potential drug treatment in gastric cancer [22] and has been trialed in other cancer treatments [23]. Nevertheless, the low bioavailability of LOV related to its poor water solubility [24,25] makes the formulation of this drug for oral administration quite challenging. Several efforts have been made to improve both the solubility and bioavailability of this drug through different approaches, including the synthesis of nanocrystals [26], nanomatrix-supported lipid bilayers [27], microspheres [28], self-nanoemulsifying drug delivery systems [29,30], methylated beta-cyclodextrin [31], solid lipid nanoparticles [32] and nanostructured lipid carriers [25]. Even though significant improvements in bioavailability have been achieved with these techniques, there are several drawbacks. In general, these strategies involve large quantities of organic solvents, additives and solubilizers that have toxic effects and increase the cost of production [26]. Limitations in drug loading and drug expulsion during storage [25] are other factors to be considered. Approaches based on particle-size reduction produce deficient mechanical properties of the material, as for example low flow and high adhesion. Furthermore, the resulting powders become extremely difficult to handle [9,24]. For an inclusion complex, a special molecular weight is required, and for emulsions, a minimum solubility of the drug in the oil phase is necessary [9,24]. Recently, solid dispersion approach has been successfully applied to improve the dissolution rate of LOV using acetylsalicylic acid as coformer [33], which has proved to be a simple strategy to overcome the poor water solubility of this drug.

**Figure 1:**
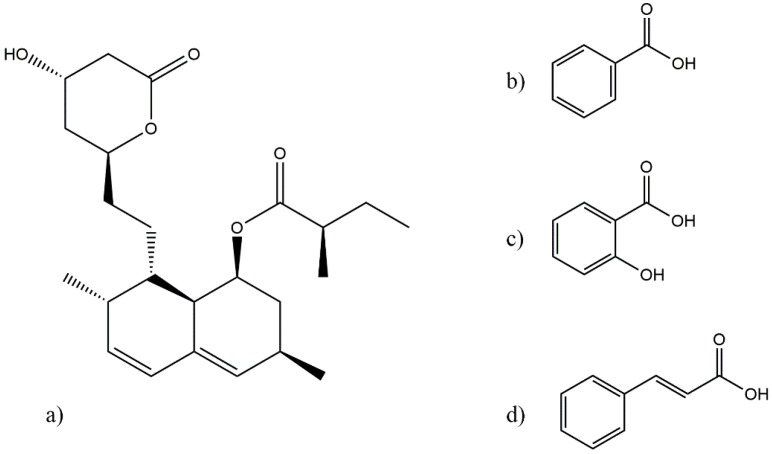
Chemical structure of (**a**) lovastatin (LOV), (**b**) benzoic acid (BEN), (**c**) salicylic acid (SAL) and (**d**) cinnamic acid (CIN).

Therefore, in this study, attempts have been made to produce eutectics of LOV with a series of water-soluble carboxylic acids. Benzoic acid (BEN), salicylic acid (SAL) and cinnamic acid (CIN) were used as coformers, which exhibit aqueous solubilities at 25 °C of 3400 mg/L [34], 2240 mg/L [35] and 546 mg/L, respectively. In addition, BEN as a food ingredient is not considered a hazard for human health [36], and SAL and CIN are permitted by the U.S. Food and Drug Administration (FDA) for direct addition to food as flavoring agents and adjuvants [37,38]. The chemical structures of LOV and the coformers are presented in Figure 1. To determine the exact eutectic composition, both binary phase and Tammann diagrams were constructed for the three systems, prepared using differential scanning calorimetry (DSC) data. The eutectics were characterized using X-ray powder diffraction (PXRD), Fourier-transform infrared spectroscopy (FT-IR) and scanning electron microscopy (SEM). Solubility assessments were applied to both the eutectic composition and a point outside of the eutectic composition for the three eutectic systems.

## 2. Materials and Methods 

### 2.1. Materials

The lovastatin (LOV) raw material was purchased from Valdequimica, Brazil (purity reported to be >99%) and used without further purification. Adipic acid (ADI, 99% purity), citric acid (CIT, 99% purity), and tartaric acid (TAR, 99% purity) were acquired from Fischer Scientific (Hampton, NH, USA). Benzoic acid (BEN, 99% purity), salicylic acid (SAL, 99% purity) and cinnamic acid (CIN, 99% purity) were purchased from Merck (Darmstadt, Hesse, Germany), Mallinckrodt (Phillipsburg, NY, USA) and Sigma-Aldrich (Saint Louis, MO, USA), respectively. Lovastatin pharmaceutical secondary standard traceable to USP and PhEur Fluka brand used in the HPLC quantification studies was purchased from Sigma-Aldrich. All solvents were HPLC/UV grade, and water was purified using a Millipore system filtered through a Millipore 0.22 µm Millipak™ 40 membrane.

### 2.2. Lovastatin Eutectic Mixture Screening

Lovastatin and the carboxylic acids adipic (ADI), citric (CIT) tartaric (TAR), BEN, SAL and CIN were individually and accurately weighed to obtain 50 mg of a binary mixture in a 1:1 molar ratio. The binary mixtures were ground together using liquid (ethanol 20 μL) assisted grinding technique. The obtained solids were evaluated in a DSC to determine the eutectic formation.

### 2.3. LOV-BEN, LOV-SAL and LOV-CIN Eutectic Systems

#### 2.3.1. Determination of Mixture Composition at the Eutectic Point

The composition at the eutectic point of LOV and the selected carboxylic acid (BEN, SAL or CIN) was obtained by the construction of Tammann and binary phase diagrams. The diagrams were prepared according to the literature [11,39], considering the recommendations given by Rycerz (2013) for the use of DSC data [39]. Different molar ratios of LOV and the selected carboxylic acids (e.g., 0:1, 0.1:0.9, 0.2:0.8, 0.3:0.7, 0.4:0.6, 0.5:0.5, 0.6:0.4, 0.7:0.3, 0.8:0.2, and 0.9:0.1) were prepared as described in the previous section. Briefly, the appropriate amount of each component to obtain 50 mg of the desired binary mixture was placed in a glass mortar and pestle. The mixture was then ground for 10 min, assisted by 20 μL of ethanol. Then, 2 mg of the obtained solid was placed into an aluminum crucible and analyzed from 40 to 200 °C at a heating rate of 10 °C/min using the DSC equipment described below. Higher and lower heating rates (e.g., 5 °C/min and 50 °C/min) were used to improve thermal events and avoid peak overlap. In the phase diagram construction, the onset temperature of the first endothermic event was used as the solidus point and the peak of the second endothermic event was considered the liquidus point. In the case of the Tammann diagrams, the onset temperature and the enthalpy of fusion of the first endothermic event were used. The analyses of data were performed using TA Instruments-Waters LLC Universal Analysis 2000 software (version 4.5A, New Castle, DE, USA, 2016). Each analysis was performed in triplicate.

#### 2.3.2. Preparation of Bulk Mixtures at the Eutectic Composition

To perform solid-state characterization and solubility assessments, the LOV-BEN, LOV-SAL and LOV-CIN eutectic systems were prepared in a larger amount in their respective eutectic composition. The same method as described in Section 2.2 was used for the scale-up. In short, the following amounts were accurately weighed: for the LOV-BEN system, 438 mg LOV and 563.7 mg BEN (43.7:56.3 wt%); for the LOV-SAL system, 662 mg LOV and 339.0 mg SAL (66.2:33.8 wt%); and for the LOV-CIN system, 308 LOV and 692.9 CIN (30.8:69.2 wt%). The components of each system were mixed and homogenized for 20 min in a glass mortar and pestle using 400 µL of ethanol as a catalyst. The obtained solids were dried at 60 °C for 1 h and stored in a desiccator until further analysis.

### 2.4. Characterization of the Eutectic Mixtures

#### 2.4.1. Differential Scanning Calorimetry (DSC) Analysis

Differential scanning calorimetry curves of the obtained solids were acquired using a DSC-Q200 (TA Instrument, New Castle, DE, USA) equipped with a TA Refrigerated Cooling System 90, using aluminum crucibles with approximately 2 mg of the sample under a dynamic nitrogen atmosphere (50 mL/min) and a heating rate of 10 °C/min in the temperature range from 40 to 200 °C.

#### 2.4.2. Powder X-ray Diffraction (PXRD) Study

Powder X-ray diffraction patterns were collected using a PANalytical Empyrean diffractometer equipped with a Cu K*α* source (*λ* = 1.5418 Å) operated at 45 kV and 40 mA. Powder samples were placed onto a zero-background sample holder. The patterns were recorded over an angular range of 4–40° (2θ) with a step size of 0.0130° and a step time of 48 s using a silicon strip detector (PIXcel 1D). The diffractograms were obtained at ambient conditions.

#### 2.4.3. Fourier Transform Infrared (FT-IR)

Fourier transform infrared spectra were recorded on a Thermo Scientific Nicolet 6700 FT-IR (Waltham, MA, USA) fitted with a diamond attenuated total reflectance (ATR) accessory. The samples were placed into the ATR cell without further preparation and analyzed in the range of 4000–600 cm^−1^, collecting 32 scans at a resolution of 4 cm^−1^.

#### 2.4.4. Scanning Electron Microscopy (SEM)

Micrographs were obtained using a Hitachi Tabletop TM3000 scanning electron microscope (Fischeln, Krefeld, Germany) operated in the range of 5–30 kV. The samples were mounted on the sample holder using carbon double-sided adhesive tape. Vacuum was reached using an oil-free system consisting of a diaphragm pump for rough evacuation and a high-performance turbo-molecular pump for main pumping.

### 2.5. Physicochemical Study

#### 2.5.1. Apparent Solubility 

The apparent solubilities of LOV and LOV eutectics were determined using the excess method in sodium lauryl sulphate (SLS) 0.25% (*w*/*v*) by oversupplying the solid to 1.5 mL of pre-equilibrated media at 37.5 °C in microtubes. The resulting slurry was maintained at constant temperature and agitation using a BIOSAN TS-100C Thermo-Shaker (Riga, Latvia) for 24 h. After this time, samples were centrifuged at 6000 rpm for 10 min in a Thermo Scientific Sorvall ST 16R centrifuge maintaining the temperature of evaluation. The samples were filtered through a 0.45 μm filter using a Sartorius stainless-steel syringe filter holder. The volume was adjusted accordingly to obtain a concentration within the analytical curve.

#### 2.5.2. Intrinsic Dissolution Rate (IDR) Determination

Intrinsic dissolution rate determination was conducted using rotating disk method according to USP30-NF25 1087 Apparent dissolution test [40]. First, 100 mg of the sample was compacted into 0.8 cm^2^ surface using a VIV TEK hydraulic press (Yangzhou, Jiangsu, China) with a manometer to 290 psi. Samples were analyzed in a SOTAX s7 dissolution test system, using 300 mL of SLS 0.25% (*w*/*v*) previously heated at 37 ± 0.5 °C as a dissolution medium and at a rotation speed of 75 rpm. Five mL of the samples were withdrawn at specific time intervals. To maintain a constant total volume a 5 mL aliquot of preheated fresh medium was replaced into the vessels. The sample aliquots were filtered using a 0.45 μm membrane placed into a Sartorius stainless-steel syringe filter holder and injected without further dilutions. The sink conditions were maintained throughout the dissolution experiment.

#### 2.5.3. High-Performance Liquid Chromatography (HPLC) Analysis

The drug concentration was determined using the HPLC pharmacopeial method [41]. A Dionex Ultimate 3000 HPLC system (Waltham, MA, USA), equipped with variable wavelength detector, pump, variable temperature compartment column and an autosampler was used. The mobile phase was composed of 65% acetonitrile and 35% water with 0.1% phosphoric acid, a flow rate of 1 mL/min, 50 μL injection volume, detection at 238 nm in a Nucleosil 100-5C18 column (250 mm × 4.0 mm, 5 μm), and a temperature of 37.5 °C were used.

A five-standards calibration curve of LOV concentrations ranging from 5 to 25 μg/mL was injected in the same sequence as the samples. The obtained regression coefficient (R^2^) was in all cases ≥0.99. Each experiment was performed in triplicate, and average values were calculated.

## 3. Results

### 3.1. Eutectic Mixture Screening

**Figure 2:**
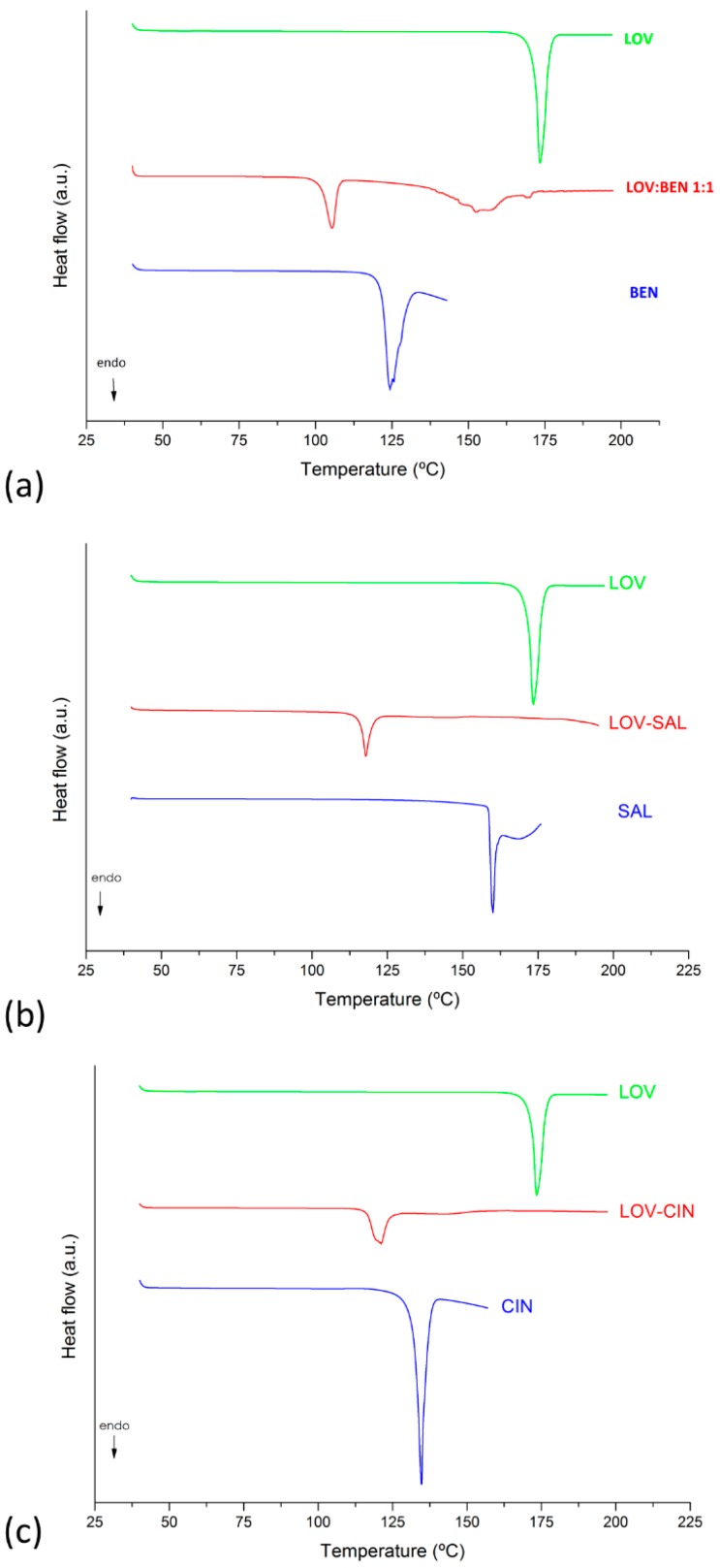
Differential scanning calorimetry (DSC) curves obtained in the eutectic mixture screening of LOV and selected carboxylic acids: (**a**) LOV, BEN and LOV-BEN, (**b**) LOV, SAL and LOV-SAL and (**c**) LOV, CIN and LOV-CIN.

Differential scanning calorimetry is the primary technique used to identify eutectic formation; hence, it was applied to screen the LOV and coformers/excipients in 1:1 binary mixture composition. Adipic acid, CIT and TAR were also evaluated and did not form eutectic mixtures with LOV. On the other hand, BEN, SAL and CIN were able to form eutectic mixtures with LOV. As expected for eutectic mixtures, a unique endothermic event is observed in Figure 2a–c corresponding to LOV-BEN, LOV-SAL and LOV-CIN eutectic formation, respectively. The thermograms show a significant reduction in the melting temperature of the eutectic systems compared to the melting temperatures of the pure compounds, confirming eutectic formation. Therefore, the composition at the eutectic point, the solid-state characterization and the solubility analyses were performed on the LOV-BEN, LOV-SAL and LOV-CIN systems.

### 3.2. Binary Phase and Tammann Diagrams

**Figure 3:**
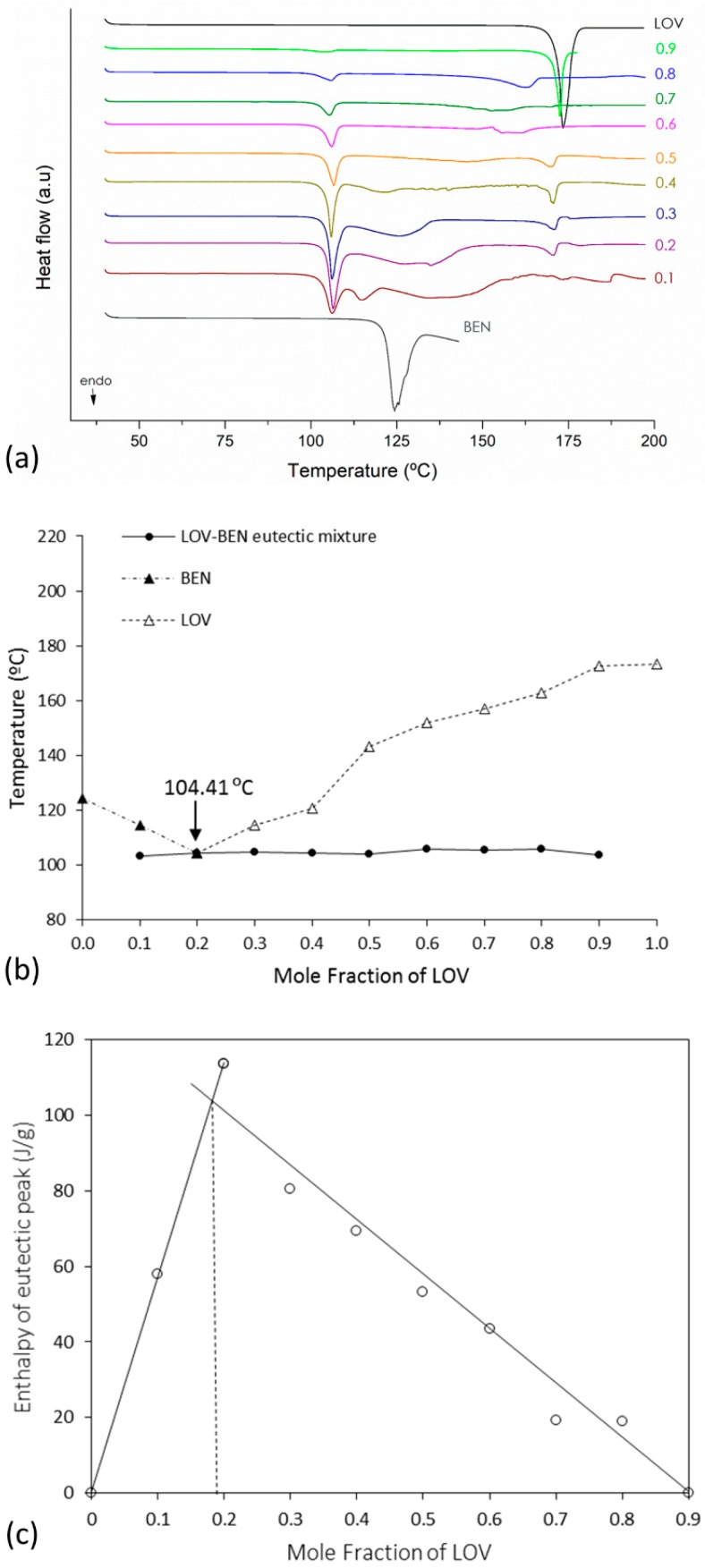
(**a**) DSC curves used to construct (**b**) the eutectic phase diagrams (▲) where (Δ) represents the variable liquidus line; (●) represents the solidus line. (**c**) The Tammann diagram of LOV-BEN of eutectic mixture.

**Figure 4:**
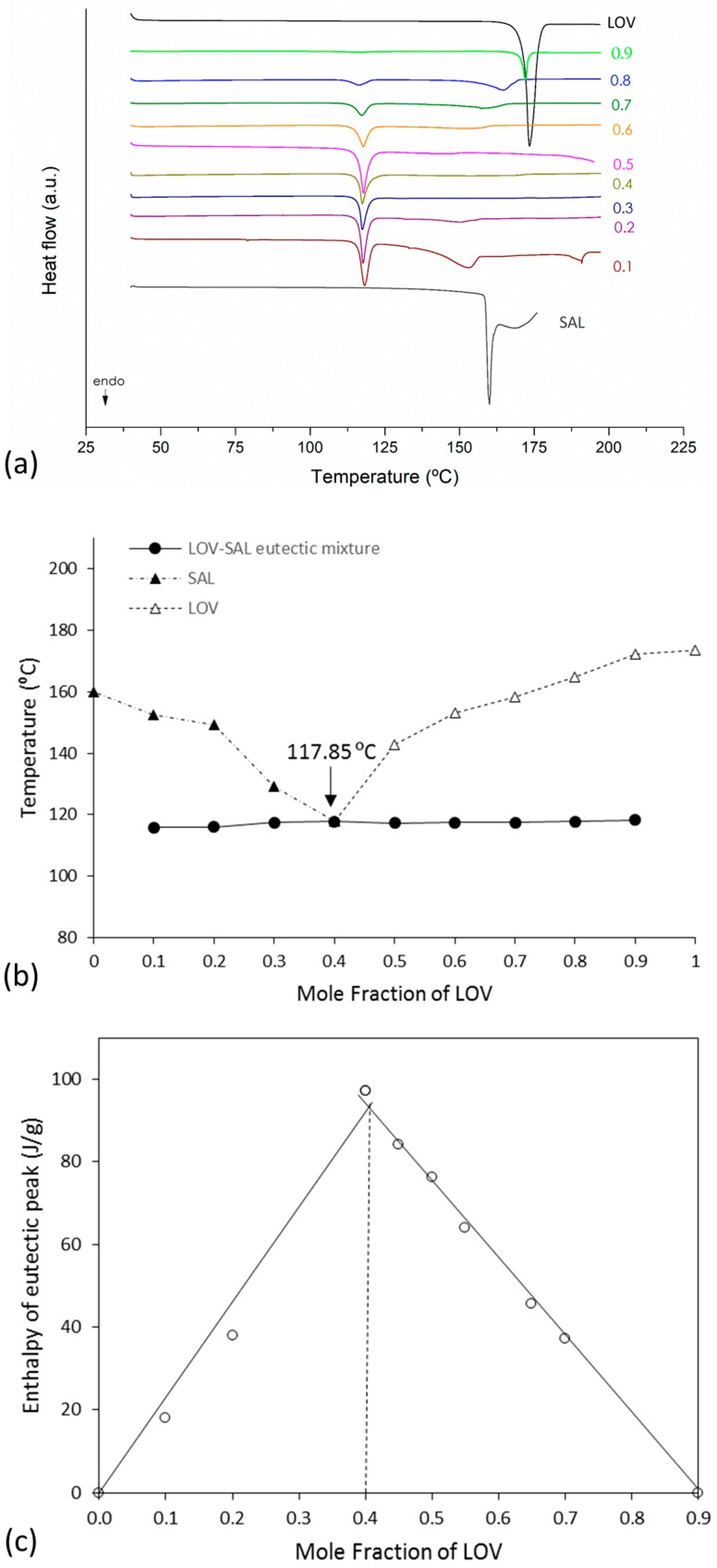
(**a**) DSC curves used to construct (**b**) the eutectic phase diagram (▲) where (Δ) represents the variable liquidus line; (●) represents the solidus line. (**c**) The Tammann diagram of LOV-SAL system.

**Figure 5:**
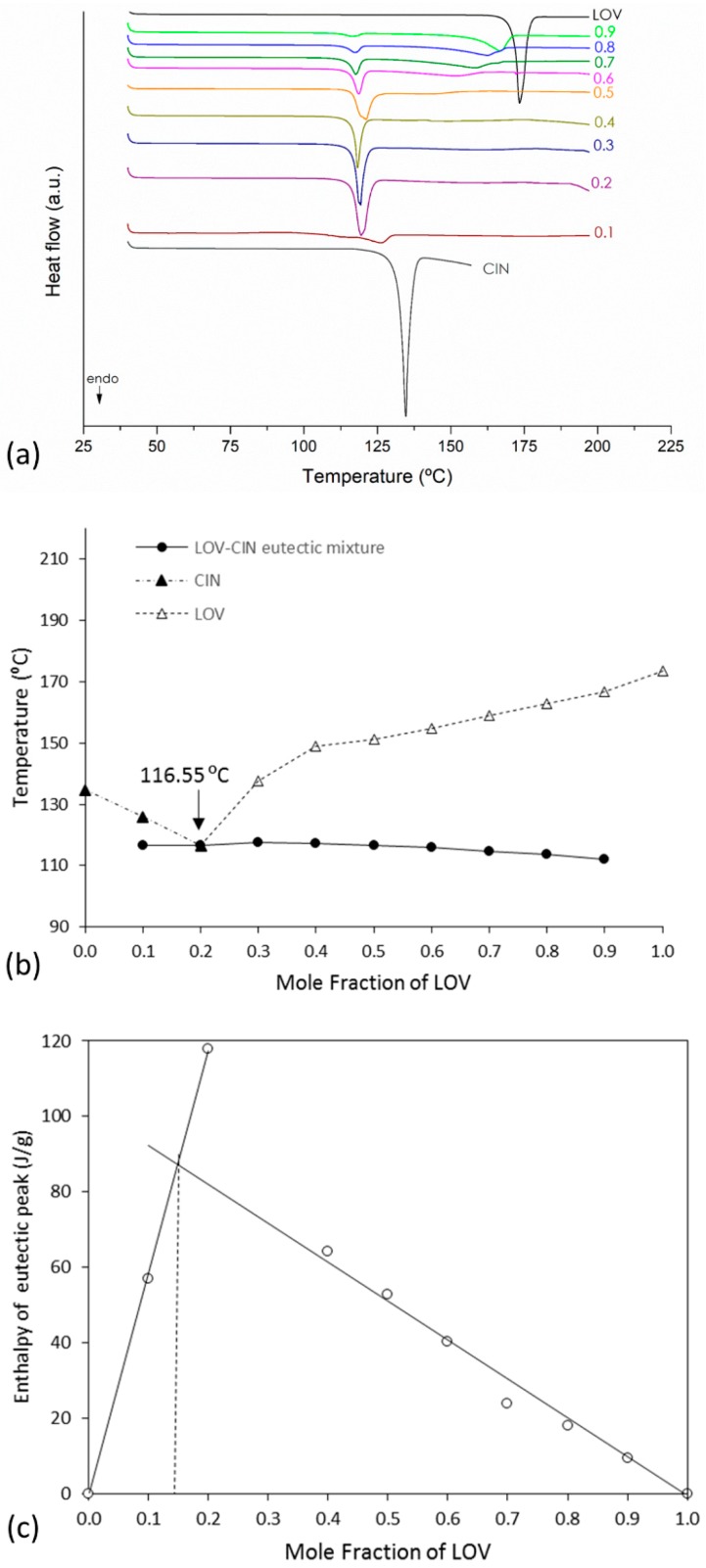
(**a**) DSC curves used to construct (**b**) the eutectic phase diagram (▲) where (Δ) represents the variable liquidus line; (●) represents the solidus line. (**c**) The Tammann diagram of LOV-CIN system.

Suitable determination of eutectic composition requires the construction of both the binary and Tammann diagrams [39]. In the phase diagram, the melting temperature of the eutectic mixture (solidus point) and the excess component (liquidus point) is plotted as a function of the mole fraction of LOV. On the other hand, the Tammann diagram shows the systematic dependence of molar enthalpy associated with the eutectic effect on the mole fraction [39]. These diagrams were constructed for the three eutectic systems, LOV-BEN, LOV-SAL and LOV-CIN. The eutectic phase diagrams were drawn using the melting endotherms of the binary mixtures obtained in variable molar ratios of LOV:coformer: 0:1, 0.1:0.9, 0.2:0.8, 0.3:0.7, 0.4:0.6, 0.5:0.5, 0.6:0.4, 0.7:0.3, 0.8:0.2, 0.9:0.1, and 1:0 mol/mol. Figure 3a,b, Figure 4a,b and Figure 5a,b present the DSC curves used to generate the diagrams (a) and the eutectic phase diagrams obtained (b) for the LOV-BEN, LOV-SAL and LOV-CIN systems, respectively. The Tammann diagrams for the LOV-BEN, LOV-SAL and LOV-CIN eutectic mixtures are shown in Figure 3c, Figure 4c and Figure 5c, respectively, where the intercept of the linear curve indicates the mole fraction of drug:coformer in the eutectic composition. Therefore, from the constructed phase and Tammann diagrams, the molar ratios of LOV-BEN, LOV-SAL and LOV-CIN were 0.19:0.81, 0.40:0.60 and 0.14:0.86, respectively.

### 3.3. Solid-State Characterization of LOV Eutectic Systems

One of the requirements for eutectic mixture formation is that the components forming the mixture should have moieties that can interact to form noncovalent interactions [4], with a major presence of electrostatic interactions [42]. In the case of LOV and the selected carboxylic acids (Figure 1), the main interaction should occur between the carbonyl and hydroxyl groups present in both LOV and the carboxylic acids used. However, according to Cherukuvada and Nangia (2014) a new chemical entity is formed when the adhesive interactions (i.e., attractive forces between different molecules) are higher than the cohesive (i.e., intermolecular forces occurring between the same molecule) [6]. In the absence of structural rules to determine whether the cohesive or adhesive forces will dominate ones over the others, the strategy is one of monitoring a new crystal phase or chemical entity formation through appropriate techniques. In this context, PXRD analyses determine crystal structure modifications and FT-IR is useful to follow changes in vibrational modes due to molecular interactions in the solid state [43]; because, it is quite sensible to crystal structure modifications, conformational rearrangements and chemical environment changes. Therefore, the purpose of this section is to show that no new chemical entity or crystalline phase has been prepared, only the eutectic systems of LOV-BEN, LOV-SAL and LOV-CIN.

#### 3.3.1. PXRD and FT-IR Analyses 

**Figure 6:**
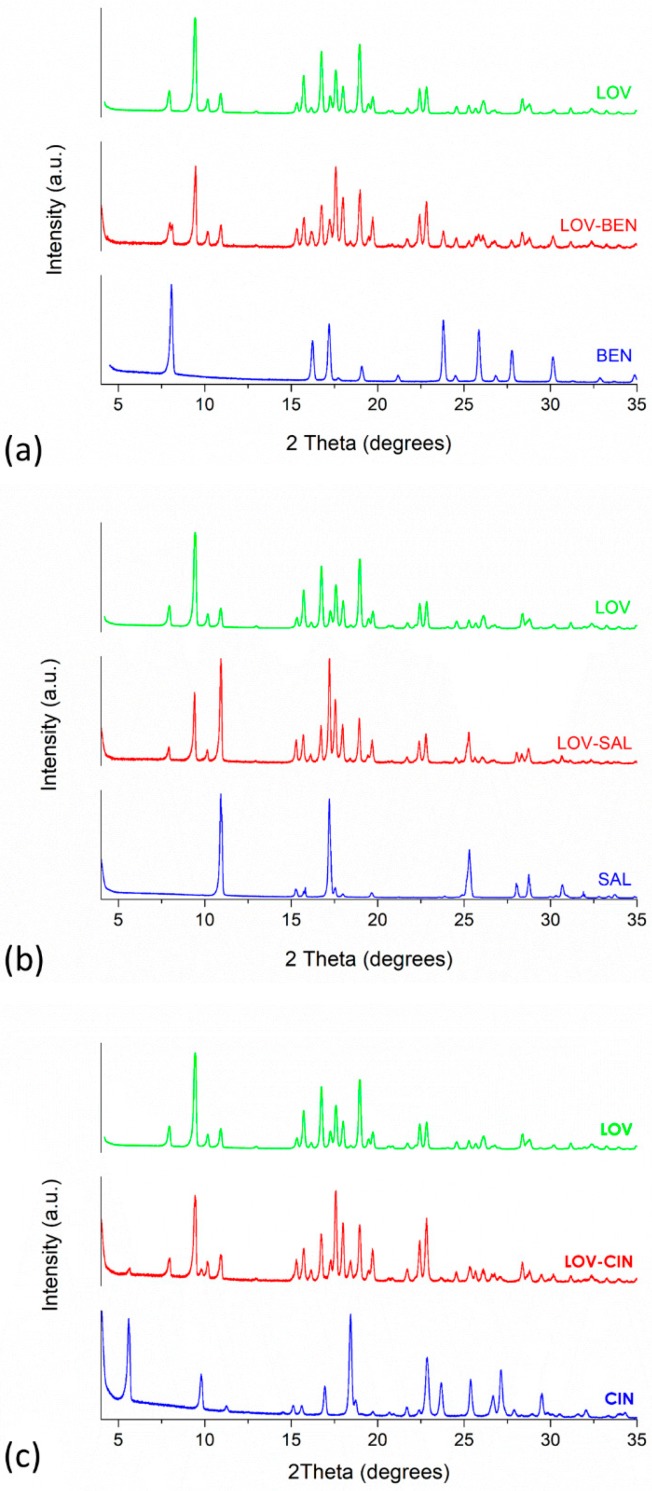
Powder X-ray diffraction (PXRD) patterns of LOV and selected carboxylic acids at the eutectic composition: (**a**) LOV, BEN and LOV-BEN, (**b**) LOV, SAL and LOV-SAL and (**c**) LOV, CIN and LOV-CIN.

Powder X-ray diffraction and FT-IR analyses were performed to determine crystalline forms as a qualitative indicator of the crystallinity of the samples and interactions presented between LOV and the selected carboxylic acids. The rise of new or shifted bands in the FT-IR spectrum is indicative of molecular interactions in the solid state [14]. In addition, new reflections in the PXRD pattern that could not be assigned to any of the pure components is evidence of modifications in the crystal structure. Figure 6 presents the PXRD patterns and Figure S1 shows the FT-IR spectra of LOV, pure BEN, SAL and CIN and their respective eutectic mixtures, LOV-BEN (a), LOV-SAL (b) and LOV-CIN (c).

Although the PXRD patterns of LOV and the selected carboxylic acids possess several superimposed reflections, it is possible to distinguish at least two characteristic reflections. In the case of BEN, the reflections at 23.79° and 27.76° are distinctive and relatively intense. In the case of the LOV-BEN eutectic mixture, considering that the mole ratio at the eutectic composition is equivalent to 43.7:56.3 wt% of the parent compounds, the reflection of both components is present in the PXRD pattern as shown in Figure 6a. It is also possible to observe the split peak approximately 8° corresponding to the near reflections of LOV at 7.94° and BEN at 8.07°.

Similarly, SAL presents two distinctive reflections at 28.01° and 30.69°. However, the mole ratio of this eutectic system is equivalent to 66.2:33.8 wt%, with a higher content of LOV, and consequently, the PXRD pattern of the LOV-SAL eutectic mixture presented in Figure 6b corresponds to mainly LOV reflections. The observed superimposed reflections of LOV and SAL at 10.92°, 17.19° and 25.30° are increased in the diffractogram of the LOV-SAL system. Cinnamic, on the other hand, presents four characteristic reflections at 5.59°, 9.80°, 23.67°, and 27.14°. In this case, the composition of the LOV-CIN system is higher in CIN (69.2 wt%), and the PXRD pattern possesses reflections of both components (Figure 6c). Finally, none of the three studied eutectic systems exhibited unassigned reflections, and therefore there is no evidence through the analyzed PXRD patterns of new crystalline phase formations.

The FT-IR of LOV shows characteristic peaks at 3535.81 cm^−1^ corresponding to the alcohol O–H stretching, at 1695.52 cm^−1^ consistent with the ester and lactone carbonyls C=O stretching, and at 1214.52 cm^−1^ corresponding to a carbon-oxygen C–O single bond asymmetric bend. The FT-IR for LOV-BEN, LOV-SAL and LOV-CIN eutectic systems show O–H stretching peaks at 3537.81, 3536.03 and 3538.24 cm^−1^, C=O stretching peaks at 1694.68, 1695.55 and 1694.99 cm^−1^, and C–O asymmetric bending at 1214.95, 1212.22 and 1216.00 cm^−1^, respectively. Therefore, no major shifting in the LOV band positions in comparison to the eutectic mixtures suggests that there are no molecular interactions in the solid state. The combination of the results from the DSC, FT-IR and PXRD experiments confirmed that a eutectic mixture was formed in the systems.

#### 3.3.2. Scanning Electron Microscopy (SEM)

**Figure 7:**
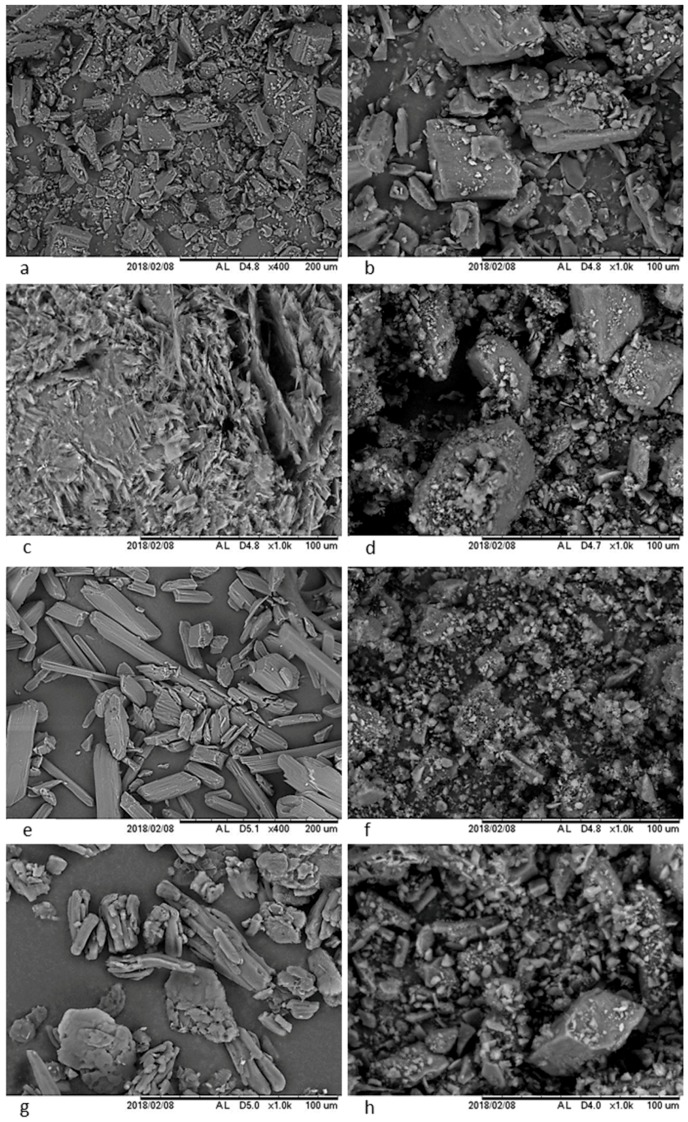
Micrographs of: (**a**,**b**) pure LOV) (**c**) pure BEN, (**d**) LOV-BEN eutectic mixture, (**e**) pure SAL, (**f**) LOV-SAL eutectic mixture, (**g**) pure CIN and (**h**) LOV-CIN eutectic mixture.

Scanning electron microscopy images accurately contrast the morphological characteristics of LOV and the selected coformers, and the micrographs are shown in Figure 7: pure LOV (Figure 7a,b), pure BEN (Figure 7c), pure SAL (Figure 7e) and pure CIN (Figure 7g). LOV-BEN (Figure 7d), LOV-SAL (Figure 7f) and LOV-CIN (Figure 7h) have significantly different morphologies compared to the individual components. A decrease in particle size related to the intrinsic characteristics of the grinding process is also evident in the three eutectic systems. 

### 3.4. Solubility Determinations of the Eutectic Systems

To evaluate the impact of eutectic mixture formation on drug solubility improvements, the apparent solubility was evaluated from two different compositions. The first was the eutectic composition obtained from the phase and Tammann diagrams. The second composition was selected by observation from the thermograms in Figure 3, Figure 4 and Figure 5, corresponding to LOV-BEN, LOV-SAL and LOV-CIN, respectively. Therefore, the composition out of the eutectic point was selected from the DSC curves showing a unique endothermic event and a straight baseline. The compositions at the eutectic point were 0.19:0.81 for LOV-BEN, 0.40:0.60 for LOV-SAL and 0.14:0.86 for LOV-CIN. In addition, the selected compositions outside of the eutectic point were 0.70:0.30 for LOV-BEN, 0.58:0.42 for LOV-SAL and 0.70:0.30 for LOV-CIN. Table 1 presents the results obtained from the apparent solubility determinations.

Eutectic materials were evaluated after solubility determinations by PXRD measurements, which are presented in Figure S2 (Supporting Information). The same PXRD pattern was observed from the eutectic before and after solubility evaluations. In the case of LOV-CIN eutectics, the disappearance of a reflection at 5.63° (2θ position) related to the CIN raw material was observed. Therefore, the PXRD patterns confirmed that no dissociation of components had occurred in the three systems. Furthermore, there was no evidence of a new phase or cocrystal formation from the starting materials or any other crystal structure modification. 

**Figure 8:**
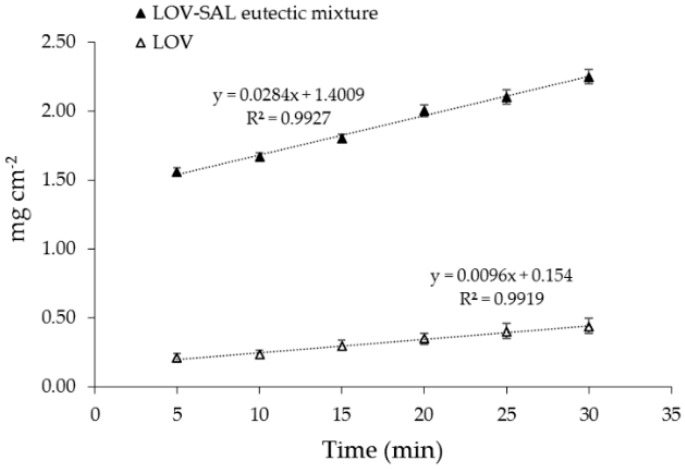
Intrinsic Dissolution Rate (IDR) profile of pure LOV and LOV-SAL eutectic mixture.

It is well known that bioavailability and the therapeutic effect of a drug depends on solubility and dissolution rate [44]. Moreover, IDR value has been demonstrated to be useful to correlate the in vivo drug dissolution dynamics [45]. Hence, the IDR was determined for the LOV-SAL eutectic system which showed to be the best solubility enhancer for LOV. Figure 8 shows a linear behavior in the intrinsic dissolution profile of pure LOV and the LOV-SAL system, with IDR values of (0.0096 ± 0.0004) mg cm^−2^ min^−1^ and (0.0284 ± 0.0002) mg cm^−2^ min^−1^, respectively, indicating that the LOV-SAL eutectic mixture showed in fact an important increment of solubility in respect to pure LOV. 

Although higher increments in LOV solubility were obtained using strategies based on nanotechnologies [27,31], it is important to stress that the eutectic formation approach is a reproducible and simple method with less expensive processing which produces an intermediary material during the formulation process. The latter conditions could be highly advantageous for pharmaceutical productions.

## 4. Conclusions

Binary eutectic mixtures of LOV with benzoic, salicylic and cinnamic carboxylic acids were able to be produced through liquid assisted grinding. Lovastatin showed improvements in solubility in all three systems. The increment of LOV solubility was found to be five-fold for the lovastatin-salicylic acid system and around four-fold for the other two systems. Further, PXRD analyses showed no dissociation of components from the eutectic system as well as no crystal structure modifications after solubility assessment. Considering the enhancement of LOV solubility, the use of carboxylic acids which are GRAS and EAFUS substances, and the accessibility of the method for the pharmaceutical industry, our results indicate that these lovastatin eutectic systems have promising applications in the preparation of new pharmaceutical formulations.

## Figures and Tables

**Table 1:** Solubility determinations of pure LOV and lovastatin eutectic systems LOV-BEN, LOV-SAL and LOV-CIN at the eutectic composition and out of the eutectic composition.

System	Composition at the Eutectic Point ^a^	Solubility (µg/mL)	Composition out of the Eutectic Point ^b^	Solubility (µg/mL)
LOV-BEN	0.19:0.81	194 ± 8	0.70:0.30	147 ± 3
LOV-SAL	0.40:0.60	259 ± 4	0.60:0.40	186 ± 5
LOV-CIN	0.14:0.86	187 ± 5	0.30:0.70	163.7 ± 0.2
Pure LOV	Solubility: 49 ± 4 ug/mL			

^a^ Eutectic composition obtained from both the Tammann and eutectic phase diagram; ^b^ Composition obtained directly by observation of the DSC curves.

## Supplementary Materials

The following are available online at https://www.mdpi.com/1999-4923/11/3/112/s1, Figure S1: FT-IR spectra of LOV and selected carboxylic acids at the eutectic composition: (a) LOV, BEN and LOV-BEN, (b) LOV, SAL and LOV-SAL and (c) LOV, CIN and LOV-CIN. Figure S2: PXRD of LOV eutectics before and after solubility determinations: (a) LOV-BEN, (b) LOV-SAL and (c) LOV-CIN eutectic systems.
