# Supplementary material for: Drug Solubility Enhancement through the Preparation of Multicomponent Organic Materials: Eutectics of Lovastatin with Carboxylic Acids
Source: Pharmaceutics. 2019 Mar 9;11(3):112. doi: 10.3390/pharmaceutics11030112 (PMC6470475; doi:10.3390/pharmaceutics11030112)
Supplement: Supplementary file 1 [file pharmaceutics-11-00112-s001.pdf]

pharmaceutics

MDPI

Supplementary Materials: Drug Solubility Enhancement through the Preparation of Multicomponent Organic Materials: Eutectics of Lovastatin with Carboxylic Acids

Andrea Mariela Araya-Sibaja, José Roberto Vega-Baudrit, Teodolito Guillén-Girón, Mirtha Navarro-Hoyos and Silvia Lucia Cuffini

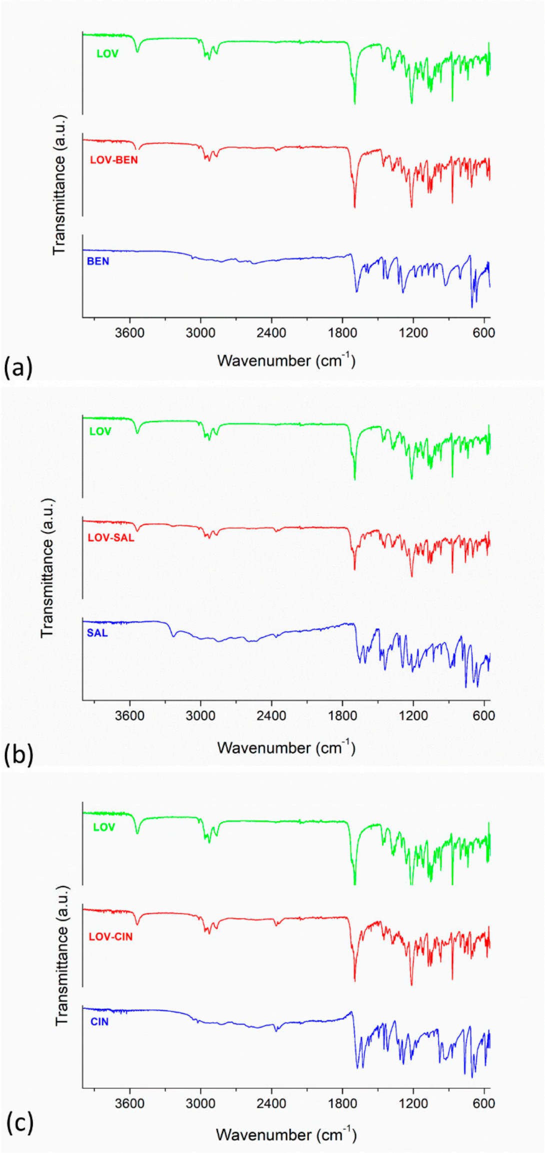

Figure S1. FT-IR spectra of LOV and selected carboxylic acids at the eutectic composition: (**a**) LOV, BEN and LOV-BEN, (**b**) LOV, SAL and LOV-SAL and (**c**) LOV, CIN and LOV-CIN.

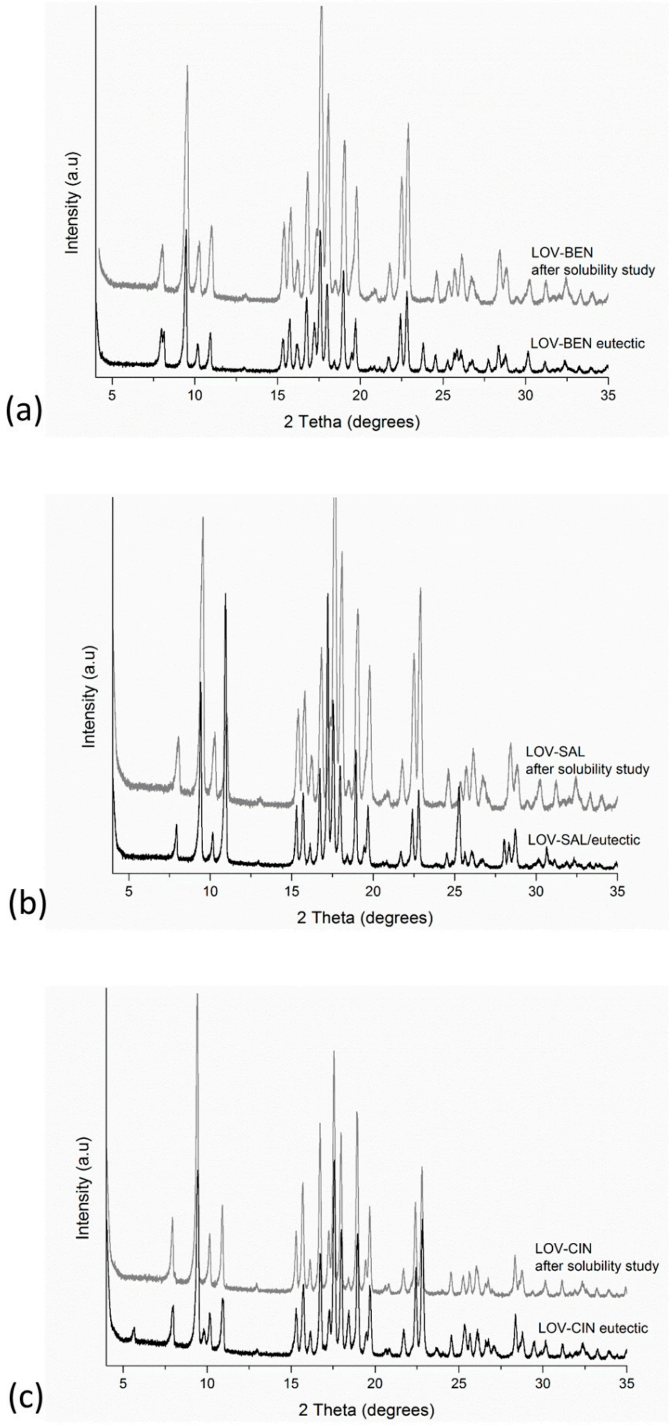

Figure S2. PXRD of LOV eutectics before and after solubility determinations: (**a**) LOV-BEN, (**b**) LOV-SAL and (**c**) LOV-CIN eutectic systems.
